# Tumor-suppressive effect of S-adenosylmethionine supplementation in a murine model of inflammation-mediated hepatocarcinogenesis is dependent on treatment longevity

**DOI:** 10.18632/oncotarget.18300

**Published:** 2017-05-30

**Authors:** Evgeniy Stoyanov, Lina Mizrahi, Devorah Olam, Temima Schnitzer-Perlman, Eithan Galun, Daniel S. Goldenberg

**Affiliations:** ^1^ The Goldyne Savad Institute of Gene Therapy, Hadassah-Hebrew University Medical Center, Jerusalem, Israel

**Keywords:** S-adenosylmethionine, methionine adenosyltransferase, Mdr2, Abcb4, hepatocellular carcinoma

## Abstract

Chronic inflammation precedes the majority of hepatocellular carcinoma (HCC) cases. We investigated the chemopreventive potential of S-adenosylmethionine (SAM), an essential donor for all methylation reactions in the cell, at the late precancerous stage of HCC development using the Mdr2-knockout (Mdr2-KO, Abcb4^−/−^) mice, a model of inflammation-mediated hepatocarcinogenesis. Previously, we revealed down-regulation of the genes regulating SAM metabolism in the liver of these mice at the precancerous stages. Now, we have supplied Mdr2-KO mice at the late precancerous stage with SAM during either a short-term (17 days) or a long-term (51 days) period and explored the effects of such supplementation on tumor development, DNA methylation and gene expression in the liver. The short-term SAM supplementation significantly decreased the number of small tumor nodules, proliferating hepatocytes and the total DNA methylation level, while it increased expression of the tumor suppressor proteins Mat1a and p21. Surprisingly, the long-term SAM supplementation did not affect tumor growth and hepatocyte proliferation, while it increased the total liver DNA methylation. Our results demonstrate that the short-term SAM supplementation in the Mdr2-KO mice inhibited liver tumor development potentially by increasing multiple tumor suppressor mechanisms resulting in cell cycle arrest. The long-term SAM supplementation resulted in a bypass of the cell cycle arrest in this HCC model by a yet unknown mechanism.

## INTRODUCTION

Hepatocellular carcinoma (HCC) is one of the most prevalent cancers in the world and is the third most frequent cause of cancer-related deaths [1]. Current treatments of HCC are of limited efficiency; moreover, HCC is much harder to treat upon its appearance rather than to its prevention, thus analysis of the precancerous stages of HCC development is of great importance for devising early diagnostics and effective preventive strategies [2].

Chronic inflammation precedes the majority of HCCs and promotes tumorigenesis by multiple molecular mechanisms which are still not well defined [3]. The Mdr2/Abcb4-knockout (Mdr2-KO) mouse is a well-known model for inflammation-mediated HCC [4] which is widely used both for studies of molecular mechanisms of HCC development [5–9] and for chemopreventive studies [10, 11]. Previously, we found in the liver tumors of Mdr2-KO/FVB mice, frequent down-regulation of transcripts of four genes that control S-adenosylmethionine (SAM or AdoMet) metabolism (Supplementary Figure 1) [12]; remarkably, two of them, Mat1a and Ahcy, were also down-regulated in a chronically inflamed mutant liver at the late precancerous stage (12 months of age) [13]. We demonstrated also a significantly decreased level of the Mat1 protein in the liver of young Mdr2-KO/FVB mice despite a normal level of the Mat1a transcript at this age [7]. Low levels of the Mat1a protein in hepatocytes should result in a reduced level of SAM which is the universal donor of the methyl group for all methylation reactions in the cell [14]. SAM is synthesized from methionine by the enzyme methionine adenosyltransferase, which is encoded by the genes MAT1A and MAT2A (Mat1a and Mat2a in mice); patients with liver cirrhosis have a 50%-reduced activity of this enzyme [15]. MAT1A is highly expressed in the adult quiescent hepatocytes, while MAT2A is expressed in fetal and in regenerating hepatocytes, and in other tissues. In human liver tumors, MAT1A expression is reduced, whereas MAT2A expression is increased; this switch facilitates cancer cell growth. In different experimental models of liver damage, both in rodents and in non-human primates, SAM administration partially restored liver function, including prevention of liver steatosis, fibrosis, and chemically induced HCC [14]. SAM was also used in human clinical trials for the treatment of alcoholic liver disease [16] and liver cirrhosis accompanied by an elevated AFP [17], both - without success, and of alcoholic liver cirrhosis with partial success [18]. The chemopreventive effect of SAM in experimental models is based on its activity as a methyl donor and on the anti-carcinogenic and anti-oxidant activities of its metabolite 5′-methylthioadenosine (MTA). MTA supplementation to Mdr2-KO mice at early age reduced liver inflammation and fibrosis [19]. In the current project, we aimed to determine whether SAM supplementation at the late precancerous stage of chronic liver disease, which is a clinically relevant state when patients seek medical help, will have a preventive effect for inflammation-mediated HCC in the murine Mdr2-KO model.

## RESULTS

### Decreased levels of transcripts encoding SAM metabolic enzymes in the chronically inflamed liver of Mdr2-KO mice

**Figure 1:**
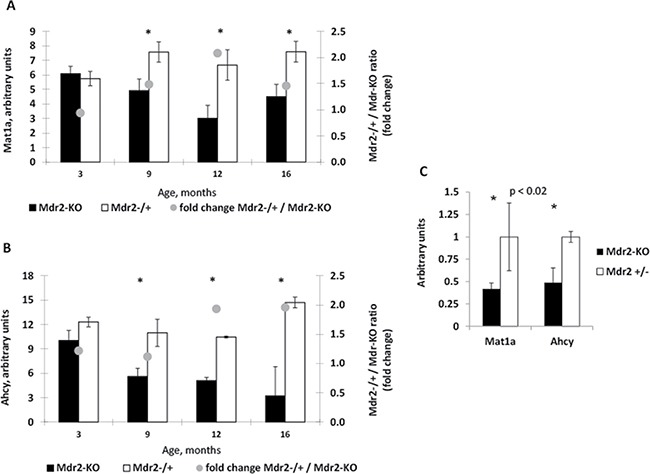
Decreased levels of the Mat1a and Ahcy transcripts in the liver of aged Mdr2-KO mouse (**A**, **B**) Dynamics of Mat1A (A) and Ahcy (B) expression in Mdr2-KO (black) compared to control Mdr2+/− (white) liver at 3, 9, 12 and 16 months of age (data from three previously published microarray datasets). (**C**) Validation of the decreased expression of Mat1A and Ahcy transcripts in the liver of Mdr2-KO mice at 12 months of age by semi-qRT-PCR; ^*^P < 0.02.

Our previous analysis of aberrant gene expression in the Mdr2-KO liver using microarrays revealed significantly decreased levels of transcripts encoding two SAM metabolic enzymes, Mat1a and Ahcy, in Mdr2-KO compared to control Mdr2+/− mice at the late precancerous [6, 13] and cancerous [12] stages; these data are summarized in Figure 1A and 1B. Remarkably, downregulation of the Mat1a and Ahcy transcripts was detected starting from the age of nine months, simultaneously with the significant increase of hepatocyte mitoses in the chronically inflamed mutant liver [13]. Now, we confirmed the down-regulation of Mat1a and Ahcy transcripts in the liver of Mdr2-KO mice at the late precancerous age (12 months) by semi-qRT-PCR (Figure 1C). Due to a key role of the methionine adenosyltransferase, encoded by the Mat1a gene, in the SAM synthesis and in the control of quiescence of mature hepatocytes [14], we tested levels of the Mat1a protein in the liver of Mdr2-KO mice at different stages of chronic liver disease.

### Decreased level and heterogeneous pattern of Mat1a protein expression at all tested stages of chronic hepatitis in Mdr2-KO mice

**Figure 2:**
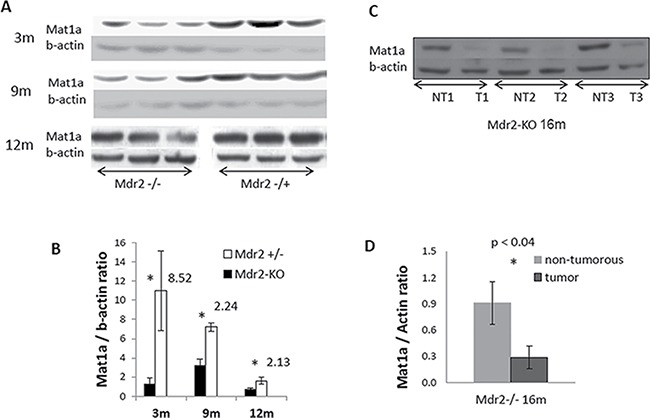
Decreased levels of the Mat1a protein in the non-tumor and tumor liver tissues of Mdr2-KO mouse revealed by immunoblotting (**A**) Mat1a immunoblotting of total liver extracts from non-tumor liver of Mdr2-KO and Mdr2+/− mice at the age of 3, 9 and 12 months. The image for 12-month-old mice was cut in the middle, and the two halves of the image were switched to fit the order of appropriate images for 3-month-old and 9-month-old mice. (**C**) Mat1a immunoblotting of extracts from three liver tumors and their matched non-tumor liver tissues of three Mdr2-KO mice at 16 months of age. (**B** and **D**) Graphical representation of the immunoblotting results shown in A and C, respectively, normalized to beta-actin levels; ^*^P < 0.04.

**Figure 3:**
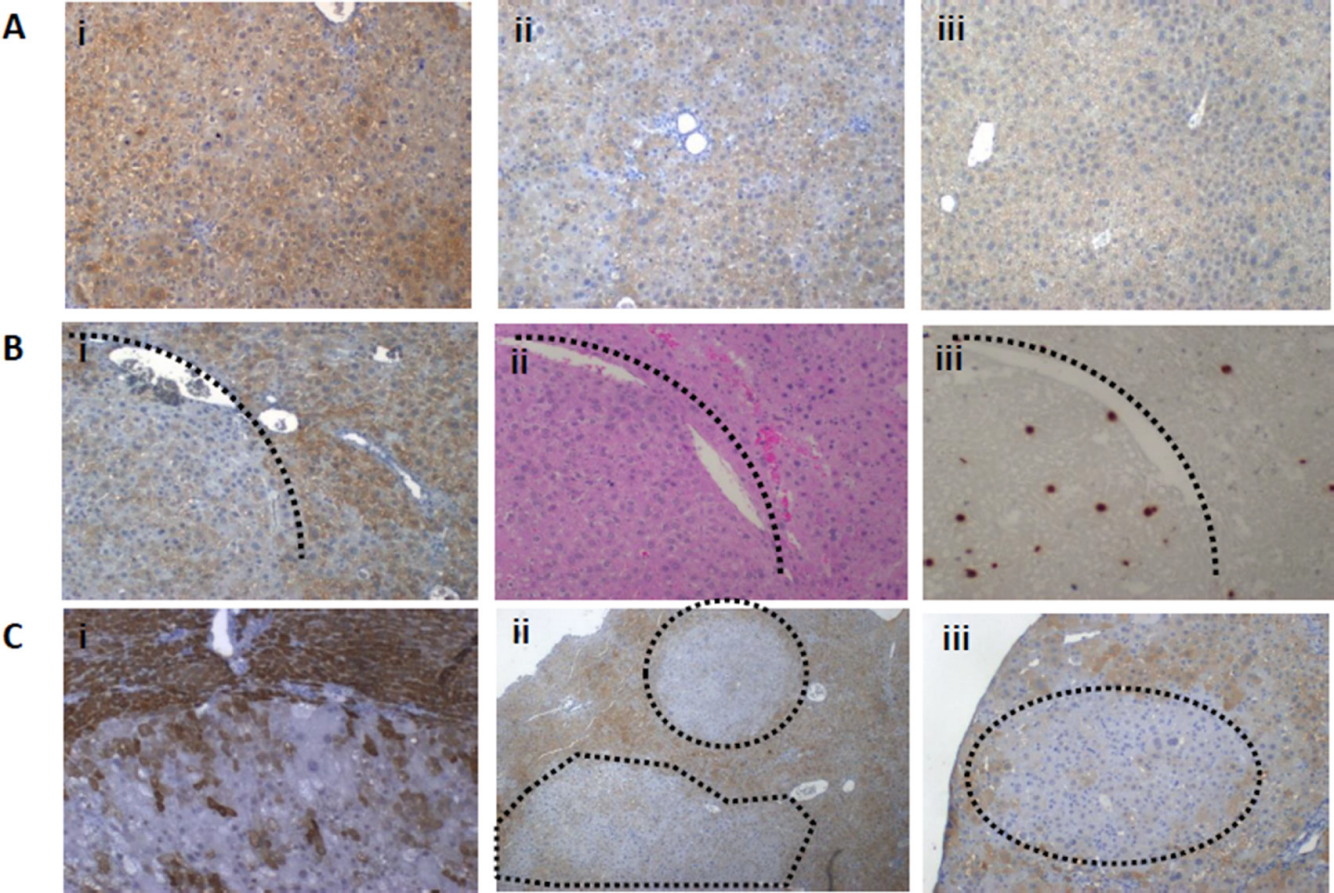
Patterns of the Mat1a protein expression in the non-tumor and tumor liver tissues of aged Mdr2-KO mouse revealed by immunohistochemical staining (**A**) Highly heterogeneous expression pattern of the Mat1a protein in the non-tumor liver tissues of Mdr2-KO mouse at the age of 9 (i) and 12 (ii) months compared to the homogenous expression pattern in the liver of 12-month-old Mdr2+/− mouse (iii). (**B**) Decreased level of the Mat1a protein in the tumor compared to surrounding non-tumor liver tissue of a 16-month-old Mdr2-KO mouse (i); the same region following H&E (ii) or BrdU (iii) staining. (**C**) Decreased level of the Mat1a protein in the tumor compared to surrounding non-tumor liver tissue of a 16-month-old Mdr2-KO mouse (i) and in the dysplastic nodules in the livers of 12-month-old Mdr2-KO mice (ii and iii).

Significantly decreased levels of the Mat1a protein in the Mdr2-KO liver were found at all tested stages of chronic liver disease (Figure 2A, 2B), including at the age of three month, when the Mat1a transcript was not down-regulated (Figure 1A). We have demonstrated previously down-regulation of the Mat1a protein in the Mdr2-KO liver of the FVB/N, but not C57BL/6, strain at the age of three months when compared to the age-matched wild type strains [7]; now we demonstrate that the Mat1a protein level is decreased in the mutant liver also when compared to the FVB/N Mdr2+/− control strain (Figure 2A). Immunohistochemical staining revealed a highly heterogeneous pattern of the Mat1a protein expression in hepatocytes of 9- and 12-month-old Mdr2-KO mice, while it was very homogeneous in the Mdr2+/− controls (Figure 3A; negative control – in Supplementary Figure 2A). The most significant down-regulation of the Mat1a protein level was observed in the majority of tested tumors and dysplastic nodules of the aged Mdr2-KO mice (Figure 2C, 2D and Figure 3B, 3C). These results are in accord with a known direct correlation between the terminal differentiation and quiescent status of hepatocytes, on the one hand, and high Mat1a levels, on the other [14].

### SAM supplementation had a significant chemopreventive effect following the short-term but not the long-term treatment

**Figure 4:**
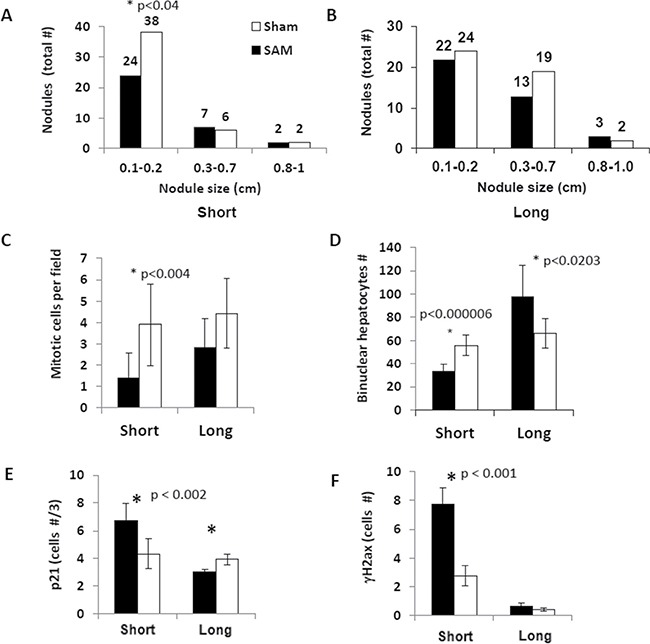
Effects of the short-term and long-term SAM supplementations on tumor load and hepatocyte proliferation in the Mdr2-KO liver Black – SAM-treated Mdr2-KO mice, white – sham-treated Mdr2-KO mice. (**A**, **B**) Number and size distribution of tumors and nodules in the livers of SAM-treated and sham-treated Mdr2-KO males following the short-term (A) and long-term (B) treatment regimens. (**C**) Number of mitotic figures in hepatocytes per 20 HPF. (**D**) Number of binuclear hepatocytes per 20 HPF. (**E**) Number of hepatocyte nuclei positive for p21 protein per 20 HPF (IHC staining). (**F**) Number of hepatocyte nuclei positive for γH2ax protein per 20 HPF (IHC staining).

To explore whether SAM supplementation as a food additive could have a chemopreventive effect at the late precancerous stage of inflammation-mediated HCC development, we supplemented 11-month-old Mdr2-KO mice with either SAM or saline by gavage during either 17 (short-term treatment) or 51 (long-term treatment) days. The short-term treatment resulted in a significant reduction of small tumor nodules and hepatocyte mitoses (Figure 4A). This was accompanied by an appropriate reduction of hepatocyte mitoses (Figure 4C and Supplementary Figure 2B) and of Ki67-positive hepatocytes (Supplementary Figure 2C), that were not accompanied by appropriate changes in BrdU inclusion into hepatocytes (Supplementary Figure 3A, 3B). Unexpectedly, however, the chemopreventive effect of SAM disappeared during its long-term supplementation (Figure 4B, 4C). The Mdr2-KO mice are characterized by a significant reduction of binuclear hepatocytes compared to the Mdr2+/− controls (Supplementary Figure 2D). The number of binuclear hepatocytes in SAM-treated compared to sham-treated Mdr2-KO mice was significantly decreased following the short-term treatment, but was significantly increased following the long-term treatment (Figure 4D). Immunohistochemical staining revealed that the short-term SAM supplementation significantly increased levels of the p21 and γH2AX proteins in hepatocytes’ nuclei of the treated Mdr2-KO mice, while the long-term SAM supplementation resulted in the decreased level of p21 in hepatocytes (Figure 4E, 4F; Supplementary Figure 3A, 3C, 3D). Neither short-term nor long-term SAM supplementation affected liver morphology, liver-to-body weight index, level of liver fibrosis (not shown), and activities of liver enzymes in the blood of Mdr2-KO mice (Supplementary Figure 4).

### The short-term SAM treatment affected the expression of specific genes that control cell cycle and inflammation

**Figure 5:**
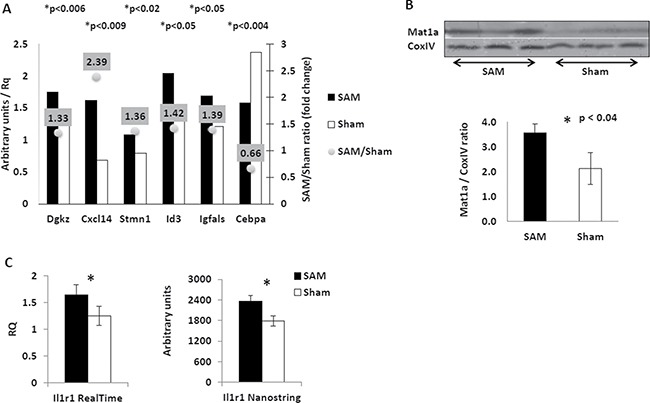
Effects of the short-term SAM supplementation on expression of selected genes at transcript and protein levels (**A**) Expression of selected genes in the liver of SAM-treated (black) and sham-treated (white) Mdr2-KO mice following short-term SAM supplementation. Semi-quantitative RT-PCR; arbitrary units normalized to expression of the Hprt gene. (**B**) Restoration of the Mat1 protein level in the liver of Mdr2-KO mice following short-term SAM supplementation. Immunoblotting of total protein extracts from the livers of SAM-treated (left triplicate) and sham-treated (right triplicate) Mdr2-KO mice. The bottom panel – quantification of the results shown in the upper panel. (**C**) Increased level of the Il1r1 transcript in the liver of SAM-treated (black) compared to sham-treated (white) Mdr2-KO mice following short-term SAM supplementation. Left panel – quantitative RT-PCR; right panel – the Nanostring test.

To further explore the molecular mechanisms of the chemopreventive effect of SAM supplementation following short-term treatment, we compared the expression of several specific genes in the liver of SAM- and sham-treated Mdr2-KO mice on the transcript and protein levels. We tested, using RT-PCR, gene expression levels of the 16 genes which previously were shown by us to be aberrantly expressed in Mdr2-KO compared to control liver at the late precancerous stage [13]. We found that only five of the 16 tested genes changed their expression following short-term SAM treatment (Figure 5A); in the untreated Mdr2-KO compared to control mice, the expression of these genes was either up-regulated about two-fold (Dgkz, Cxcl14, Stmn1, Id3), or down-regulated about two-fold (Igfals) [13]. Interestingly, in all but one case (Igfals), SAM treatment did not reverse, but further increased the aberrant expression of these genes (Figure 5A). As a non-direct test of the Igfals protein activity, we measured by RT-PCR expression levels of the Igf1 and Igfbp3 genes that are known to be down-regulated when gene Igfals is mutated [20] in the liver of the short-term SAM-treated and control Mdr2-KO mice, but found no between the groups (data not shown). Other genes, tested by either semi-qRT-PCR (Supplementary Figure 5) or by qRT-PCR (Bzw2, Hnf4a, Crem, Per3; data not shown), were similarly expressed between the two groups (genes Egfr and Crem were tested by both semi-qRT-PCR and qRT-PCR). Based on the known effect of the exogenous SAM on induction of the GADD45β expression [21] that inhibits proliferation of HCC cells during acute ischemia-hypoxia [22], we tested the expression of the Gadd45β transcript: however, it was similar in the livers of both SAM-treated and sham-treated mice (not shown).

Taking into account a wide effect of the Id3 protein on the transcriptional regulation of multiple genes, we tested whether SAM supplementation affected expression of the two well-known Id3 targets: Cdkn1b [23] and Il6 [24]. Both of these genes were equally expressed in the livers of SAM- and sham-treated Mdr2-KO mice (not shown), indicating that, most probably, the Id3 protein activity in both groups was equal. In addition, we also tested the expression of the Cebpa transcript due to the well-known suppressive role of the Cebpa protein in liver proliferation and tumorigenesis [25, 26]. We observed a 34% reduction of the Cebpa transcript level following the short-term SAM supplementation (Figure 5A). Remarkably, we also found that the short-term SAM supplementation completely restored the level of the Mat1a protein in the Mdr2-KO liver (Figure 5B), while it did not change the levels of the Cxcl14 and Stmn1 proteins (despite up-regulation of their transcripts) and the phospho-Jnk protein (not shown).

In order to reveal whether the short-term SAM treatment affected the formation of a pro-tumorigenic microenvironment in the murine liver, we compared the transcript expression of 46 genes that control this process in SAM-treated and control sham-treated Mdr2-KO mice using the Nanostring assay (Supplementary Table 1). Among all tested genes, only Il1r1, encoding receptor one of the intreleukine-1, was differentially expressed between experimental groups; the up-regulation of the Il1r1 expression in the mutant mouse liver following the short-term SAM treatment was confirmed also by qRT-PCR (Figure 5C).

### The short-term and the long-term SAM supplementation regimens had opposite effects on global DNA methylation in the Mdr2-KO liver

**Figure 6:**
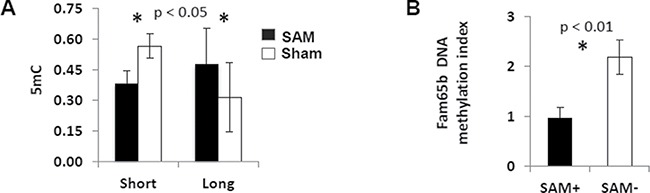
Effects of SAM supplementation on DNA methylation (**A**) Total methylation level of liver DNA was decreased following the short-term SAM supplementation, while increased following the long-term SAM supplementation (black – SAM-treatment; white – sham-treatment). (**B**) The short-term SAM supplementation reversed DNA methylation level of the CpG island in the Fam65b gene to its normal level in a healthy mouse.

Using an ELISA-based method for quantification of DNA methylation, we demonstrated that the short-term SAM supplementation resulted in a significantly decreased global liver DNA methylation, whereas the long-term SAM supplementation resulted in a reversed effect (Figure 6A). Previously, we demonstrated that chronic liver inflammation in Mdr2-KO mice at the late precancerous stage did not change global DNA methylation level, but resulted in hyper-methylation of a specific set of CpG islands, compared to Mdr2-heterozygous healthy controls [27]. Now, we tested the effect of the short-term SAM supplementation on DNA methylation in 11 of the CpG islands that were hyper-methylated in the Mdr2-KO liver, and revealed that SAM treatment reversed the methylation state of only one CpG island, located in the gene Fam65b, while it did not affect others (Figure 6B and Supplementary Figure 6). However, the expression level of the Fam65b gene in the liver was not affected by this decrease of CpG island methylation following the short-term SAM treatment (not shown).

## DISCUSSION

Encouraged by our previous finding of the reduced expression levels of enzymes regulating SAM metabolism in the liver of the Mdr2-KO mouse model of inflammation-mediated HCC [12], similarly to human HCC, in the current study, we tested the chemopreventive potential of SAM supplementation during the late precancerous stage of HCC development in this mouse model. SAM is a known chemopreventive agent in models of liver steatosis, fibrosis, and chemically induced HCC [28, 29]. Here, we demonstrate that the short-term, but not the long-term, SAM supplementation to FVB/N Mdr2-KO mice starting at the late precancerous stage, significantly decreased the numbers of small tumor nodules, of proliferating and polyploid (binuclear) hepatocytes, and the total DNA methylation level, while it increased the expression of the Mat1a, p21/Cdkn1a and γH2AX proteins in the livers of treated Mdr2-KO mice. However, the long-term SAM supplementation did not affect tumor growth and hepatocyte proliferation, while it increased the total liver DNA methylation and the number of polyploid (binuclear) hepatocytes, while it decreased the level of the p21/Cdkn1a protein in the liver.

Success of the short-term and fault of the long-term SAM administration in our study, although surprising, is not unique in translational research. Inflammation per se has different short-term and long-term effects: while acute inflammation has mainly therapeutic effects, chronic inflammation is linked with multiple chronic diseases, including cancer [30]. In the drug-mediated therapy, the long-term drug administration may be either less effective than the short-term administration [31, 32], or may have even adverse effects that were absent in the short-term administration [33–35]. The therapeutic or chemopreventive effects of SAM in animal models of liver diseases were demonstrated mainly in the short-term treatments, and when it was active in a long-term treatment of HCC – the disease was not associated with chronic inflammation, but was caused by a chemical carcinogen [36]. Nevertheless, a long-term SAM treatment (61 days) was tumor-suppressive in a mouse model of inflammation-induced colon cancer [37]. Remarkably, in both human clinical trials that lasted 24 weeks, SAM supplementation did not improve any tested parameter (doses 1.2 g/day [16] and 2.4 g/day [17]), while in the two-year trial, SAM improved survival or delayed liver transplantation only in patients with less advanced liver disease (dose 1.2 g/day [18]). These results demonstrate that SAM supplementation may have a therapeutic effect only during the early stages of chronic hepatitis. Due to the absence of the changes in the number and sizes of large tumor nodules between experimental groups in our experiments, we speculate that the short-term SAM supplementation affected mostly nodules which started to develop during the treatment period. A similar conclusion on the SAM effectiveness only in HCC establishment was done previously based on the results of SAM supplementation (11 and 24 days) in a rat model of HCC [38].

In the short-term treatment of Mdr2-KO mice, SAM efficiently inhibited hepatocyte proliferation. The inhibitory effect of SAM on hepatocyte proliferation is well known: quiescent hepatocytes have high SAM level, while proliferating hepatocytes have low SAM level [39, 14]. Exogenous SAM administration inhibits DNA synthesis in hepatocytes and HCC cells by several mechanisms including blockage of the HGF-mediated activation of LKB1/AMPK signaling [40], restoration of the DNA methylation level [41] and induction of the GADD45β expression [21, 22]. Interestingly, inhibition of hepatocyte proliferation in the Mdr2-KO liver following the short-term SAM supplementation was not accompanied by a reduction of the BrdU inclusion, but was accompanied by an increased level of γH2ax in hepatocytes (Figure 4F, Supplementary Figure 3). This may indicate the presence of stalked replication forks and an intensive DNA repair process in the SAM-treated liver [42].

Our analysis of the molecular mechanisms of the inhibitory effect of short-term SAM supplementation on hepatocyte proliferation revealed up-regulation of the tumor suppressor proteins Ma1a and p21/Cdkn1a. Silencing of tumor suppressor genes by aberrant methylation is a well-known molecular mechanism of HCC development [43, 44], which can be alleviated by demethylating agents [45]. We demonstrated down-regulation of the Mat1a protein level in the liver of Mdr2-KO mice at all tested ages (and, especially, in dysplastic nodules and tumors), confirming our previous findings for the Mat1a transcript [12, 13]. Starting from the age of 9 months, decreased level of the Mat1a protein may be explained by a decreased level of its transcript (Figure 1A), while at the age of 3 months, it may be explained by a decrease of either Mat1a translation, or its stability. Restoration of the Mat1a protein level in the Mdr2-KO liver following short-term SAM supplementation is in accord with the known ability of SAM to increase MAT1A mRNA stability [46]. High Mat1a, as well as high SAM level, is a marker of quiescent differentiated hepatocytes [14]; thus, Mat1a can be considered as a classical tumor suppressor in HCC [47]. Although p21 and Cebpa proteins both were traditionally considered as tumor suppressors [48], [49], recent new evidences demonstrate that in some cases they may be pro-oncogenic as well [50–52]. Thus, in mouse HCC models, including the Mdr2-KO model, it was shown that p21 either suppressed or promoted HCC development depending on either strong or moderate liver injury, respectively [53, 54]. Remarkably, very recently, it was demonstrated that chronic expression of p21 may result in the stabilization and accumulation of replication licensing factors inducing by this replication stress, re-replication, DNA damage and genomic alterations through error-prone DNA repair resulting in atypical p21-overexpressing highly aggressive cancer cells [55]. As it was mentioned above, indications of replication stress were present in the SAM-treated livers of Mdr2-KO mice. Similarly, the CEBP/a protein is up-regulated in a significant subset of HCC tumors and has growth-promoting activities in HCC cells [52]. On the other hand, liver tumors may escape the CEBP/a growth inhibitory activity by activation of PI3/Akt [56].

In order to further explore the molecular mechanisms of the chemopreventive effect of the short-term SAM supplementation, we tested whether it reversed the aberrant gene expression and aberrant CGI methylation that we have revealed previously in this HCC model at this late precancerous stage [13, 27]. Among the 16 genes which were aberrantly expressed in the Mdr2-KO liver specifically at this precancerous stage, only five changed their expression after SAM treatment; however, SAM did not reverse, but rather worsened their aberrant expression. Testing of the 10 among 30 inflammation-specific aberrantly methylated CGIs demonstrated that SAM supplementation did not change their DNA methylation levels, excluding reversion to the normal level of the gene Fam65b. Nevertheless, this reversion of methylation did not change the Fam65b expression. Thus, the short-term SAM supplementation did not reverse multiple aberrations of liver gene expression and the methylation caused by the Mdr2-KO mutation at the late precancerous stage.

The global hypomethylation of the liver DNA following the short-term SAM treatment of Mdr2-KO mice (Figure 6A) was surprising, because exogenous SAM supplementation was known to restore both the endogenous SAM level and DNA methylation level of hypomethylated pre-neoplastic and neoplastic liver lesions [41, 57]. Similarly, the livers of Gnmt-KO mice were characterized by high SAM level and DNA hypermethylation [58], and, in addition, it was demonstrated that SAM inhibits active demethylation of DNA [59]. Importantly, we have demonstrated previously that chronic inflammatory disease does not affect the global liver DNA methylation in Mdr2-KO mice at this age [27]; thus, the observed decrease of liver DNA methylation following the short-term SAM treatment was not a compensatory phenomenon. Liver DNA hypomethylation promotes hepatocarcinogenesis [60] and is frequently observed in dysplastic and neoplastic liver lesions appearing in mouse and human HCC development, where it is associated with poor HCC prognosis [61]. Thus, DNA hypomethylation could be one of the inhibitory mechanisms of hepatocyte proliferation during the short-term SAM supplementation, on the one hand, but could have a role in HCC progression during the long-term SAM treatment in the Mdr2-KO model, on another hand. Mechanistically, DNA hypomethylation could be caused by a combination of the known up-regulation of the Gnmt protein level by chronic SAM supplementation [38] and by a reduced Ahcy level in the liver of Mdr2-KO mice at the late precancerous stage (Figure 1C). These events should result in a significantly increased level of S-adenosylhomocysteine (SAH; Supplementary Figure 1) in the diseased liver, and SAH is a potent inhibitor of transmethylation reactions [14] known to cause DNA hypomethylation followed by DNA damage in hepatic cells [62]. Hepatocellular SAM concentration and the SAM:SAH relative level can influence diverse pathophysiological processes including tissue oxidative stress, mitochondrial function, hepatocellular apoptosis and malignant transformation [63]. Together with the known facts that both SAM deficiency (in the Mat1a-KO mice) and its increased level (in the Gnmt-KO mice) cause HCC development [58, 64], our data demonstrate that a balanced hepatic SAM level and SAM:SAH ratio are necessary to maintain liver health and prevent HCC development in chronic liver injury.

In conclusion, we have shown that SAM supplementation at the late precancerous stage in the murine model of inflammation-mediated HCC development had a chemopreventive potential only during a short-term period. Thus, the results of other short-term chemopreventive experiments *in vivo* should be taken with great precaution. We hypothesize that the short-term SAM supplementation decreased liver tumor development due to a temporal cell cycle arrest by an increased expression of several tumor suppressor genes, stalked replication forks and a global DNA hypomethylation. However, some of these effects (e.g., DNA hypomethylation), together with yet unknown factors resulted in a bypass of the cell cycle arrest during the long-term SAM supplementation. Further studies are required to understand the pathways of SAM activity and its clinical significance in primary liver cancer. The dose and time schedule of SAM supplementation at the late precancerous stage of inflammation-mediated HCC should be further investigated in combination with a deeper analysis of all other enzymes involved in SAM metabolism.

## MATERIALS AND METHODS

### Mice

All animal experiments were performed according to national regulations and guidelines of the Institutional Animal Welfare Committee (NIH approval number OPRR-A01-5011). Mice obtained a regular diet and drinking water ad libitum and under controlled conditions (22°C, 55% humidity, and 12-hour day-night rhythm). Only males were used in this study. The FVB.129P2-Abcb4^tm1Bor^ (Mdr2-KO) and wild type FVB/NJ mice were purchased from the Jackson Laboratory (Bar Harbor, ME); the control Mdr2^+/−^ heterozygotes were produced by breeding the Mdr2-KO males with FVB/NJ females. Harvesting of mouse liver tissue was done as described previously [13]. Eleven- or 10-month-old Mdr2-KO mice were gavaged daily for either 17 or 51 days, respectively, with S-adenosylmethionine (SAM; Sigma Chemicals, St Louis, MO, USA; 50 mg/kg mouse weight/day). SAM was dissolved in sterile distilled water to 10x stock solution, filtered and kept frozen at −80°C. Before the experiments, the stock solution was diluted in sterile distilled water 10-fold, and mice were gavaged with 0.2 ml of the 10 mg/ml SAM solution. Control Mdr2-KO mice were gavaged in parallel with water. At the last gavage day, in the morning, mice were injected with BrdU and after 3 h weighed and euthanized; blood was collected by orbital bleeding and the livers were removed.

### Serum analysis

The activities of liver enzymes (ALT, Alanine aminotransferase and ALP, Alkaline Phosphatase) in serum were determined using the Reflotron^®^ system and its kits (Roche, Mannheim, Germany) according to the manufacturer's instructions.

### Liver total DNA and RNA

Total liver DNA and RNA was extracted from frozen liver tissue specimens as previously described [12]. The DNA and RNA concentrations were determined by Nanodrop ND-1000 (Thermo Fisher Scientific, Wilmington, DE, USA).

### DNA methylation measurements

Global levels of 5 mC in total liver DNA (200 ng) were measured by the MethylFlash^™^ Methylated DNA Quantification Kit according to manufacturer's protocols (Epigentek, Brooklyn, NY). Methylation-sensitive restriction enzymes (MSRE) PCR has been performed as described previously [27].

### RT-PCR

The cDNA synthesis, semi-quantitative and quantitative (real-time) RT-PCR was done as described previously [13, 27]. Primers used for gene expression and DNA methylation analyses are listed in the Supplementary Table 2.

### NanoString gene expression analysis

NanoString nCounter gene expression assay was performed using two specific probes (capture and reporter) for each gene of interest. In brief, 200 ng of total RNA per sample were hybridized with customized Reporter CodeSet and Capture ProbeSet according to the manufacturer's instructions (NanoString Technologies, Seattle, WA, USA), for direct labeling of mRNAs of interest with molecular barcodes without the use of reverse transcription or amplification. Then, the hybridized samples were recovered with the NanoString Prep Station and the mRNA molecules counted with the NanoString nCounter. The resulting counts were corrected by subtracting the average value of the negative control (alien probes from the CodeSet, lacking spiked transcript) from the raw counts obtained for each RNA. The corrected raw data were finally normalized using Arl6ip1, Polrmt, Ppia, and Rps20 as housekeeping genes [65].

### Immunoblotting

Liver tissue extracts for immunoblotting were prepared by homogenization using tissue-lyzer LT (Qiagen, Germany) for 5 min at speed 40 in 600 μl of the ice-cold protein lysis buffer (50 mM Tris pH 7.6, 150 mM NaCl, 20 mM MgCl2, 1% NP40, 1 mM DTT, 5% glycerol, 1 mM PMSF and 2.5 μl/ml of protease inhibitor cocktail). After 30 min rotation at 4^°^C and 20 min centrifugation at 4^°^C, 20,800 g, the aqueous supernatant was taken to determine protein concentration by the Bradford assay. The samples were mixed with gel loading buffer (1:6), boiled, aliquoted and stored at −80^°^C until use. Immunoblotting was performed as described previously [7]. Antibodies used for immunoblotting and immunochemistry are listed in the Supplementary Table 3.

### Immunohistochemical staining

Liver tissue paraffin embedded sections were mounted on glass slides, deparaffinized and graduated with ethyl alcohol, as follows: Xylene × 2, ethanol-100% × 2, ethanol-95% × 1, ethanol-80% × 1, ethanol-60% × 1 ethanol-30% × 1 and TBS × 1 (5 min every step). Before overnight incubation with Mat1a (1/100), p21 (1/25) or γH2AX (1/1000) antibodies, antigen retrieval was done with 0.01M citrate buffer (pH 6.0) in a microwave (100% power for 13 min followed by 60% for 20 min) using a pressure cooker. The slides were washed 3 times with PBS and then labeled with a secondary HRP-conjugated streptavidin antibody. Color development with diaminobenzidine was done using the Zymed kit followed by Haematoxylin counterstaining immersed with Haematoxylin (1 min), rinsed in tap water and in order to develop the stain, left for an additional 2 min. Rehydration and clearing were done inversely to deparaffinization and dehydration. The slides were covered with a cover slip and analyzed under the Nikon Eclipse E600 light microscope equipped with an Olympus DP71 camera and Cell'A software.

### Statistical analysis

In this study, the *in vitro* experiments were performed at least 3 times and in triplicates. In the *in vivo* experiments, at least three mice per group were used. All parameters were evaluated with the two-tailed unpaired unequal variance *t*-test. Statistical evaluation of differential expression between the experimental groups was performed using the ANOVA test. A *p* value of 0.05 and less was considered significant. The data are expressed as mean ± standard deviation (SD).

## SUPPLEMENTARY MATERIALS FIGURES AND TABLES






