# Supplementary material for: Tumor-suppressive effect of S-adenosylmethionine supplementation in a murine model of inflammation-mediated hepatocarcinogenesis is dependent on treatment longevity
Source: Oncotarget. 2017 May 30;8(62):104772–84. doi: 10.18632/oncotarget.18300 (PMC5739599; doi:10.18632/oncotarget.18300)
Supplement: Supplementary file 1 [file oncotarget-08-104772-s001.pdf]

Tumor-suppressive effect of S-adenosylmethionine supplementation in a murine model of inflammation-mediated hepatocarcinogenesis is dependent on treatment longevity

Supplementary Materials

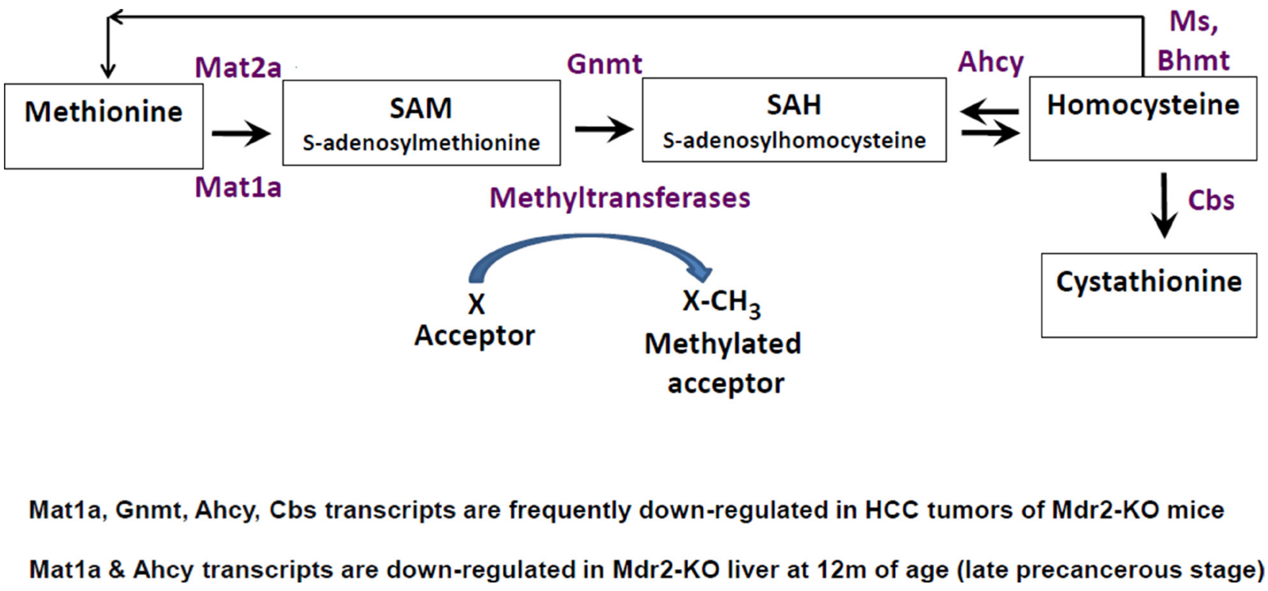

Supplementary Figure 1: Scheme of SAM metabolism. Below the scheme – genes of SAM metabolism that were previously shown by us to be down-regulated in Mdr2-KO liver at precancerous or cancerous stages.

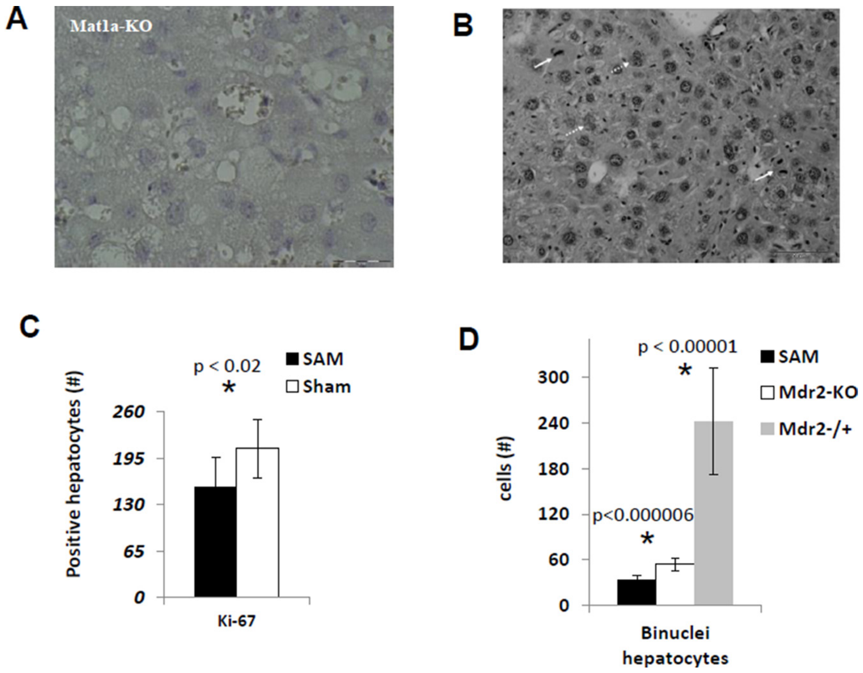

Supplementary Figure 2: Histological and IHC controls in the liver of Mdr2-KO and Mat1a-KO mice. (**A**) Negative staining control of the IHC using the anti-Mat1a antibody in the liver of 14-month-old Mat1a-KO mouse (magnification ×200). (**B**) Mitotic figures (solid arrows) and binuclear hepatocytes (dashed arrows) in the liver of 12-month-old Mdr2-KO mouse (magnification ×100). (**C**) Similar levels of K67-positive hepatocytes in SAM-treated and sham-treated mice following the short-term SAM supplementation (quantification of the IHC results). (**D**) Number of binuclear hepatocytes per 20 HPF in SAM-treated compared to sham-treated (following the short-term SAM supplementation) and control healthy Mdr2+/− mice.

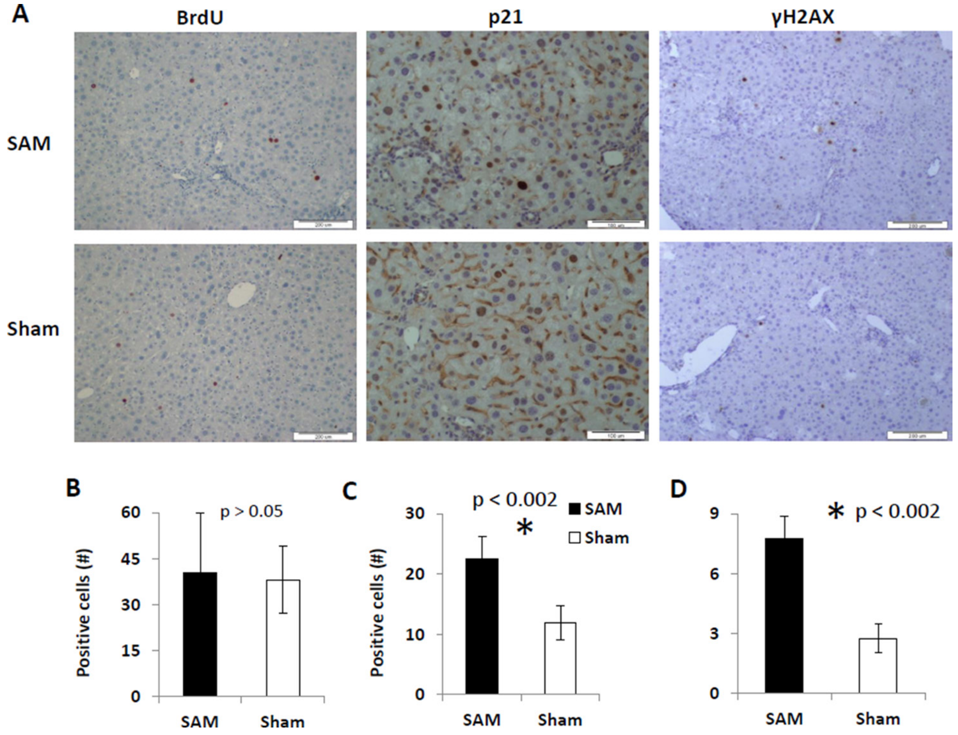

Supplementary Figure 3: IHC staining of the livers of Mdr2-KO mice following the short-term SAM- or sham-treatment. (**A**) Representative examples of staining for BrdU (quantification – in **B**), p21 (quantification – in **C**), and γH2AX (quantification – in **D**). Ten males per experimental group; $^*P < 0.002$.

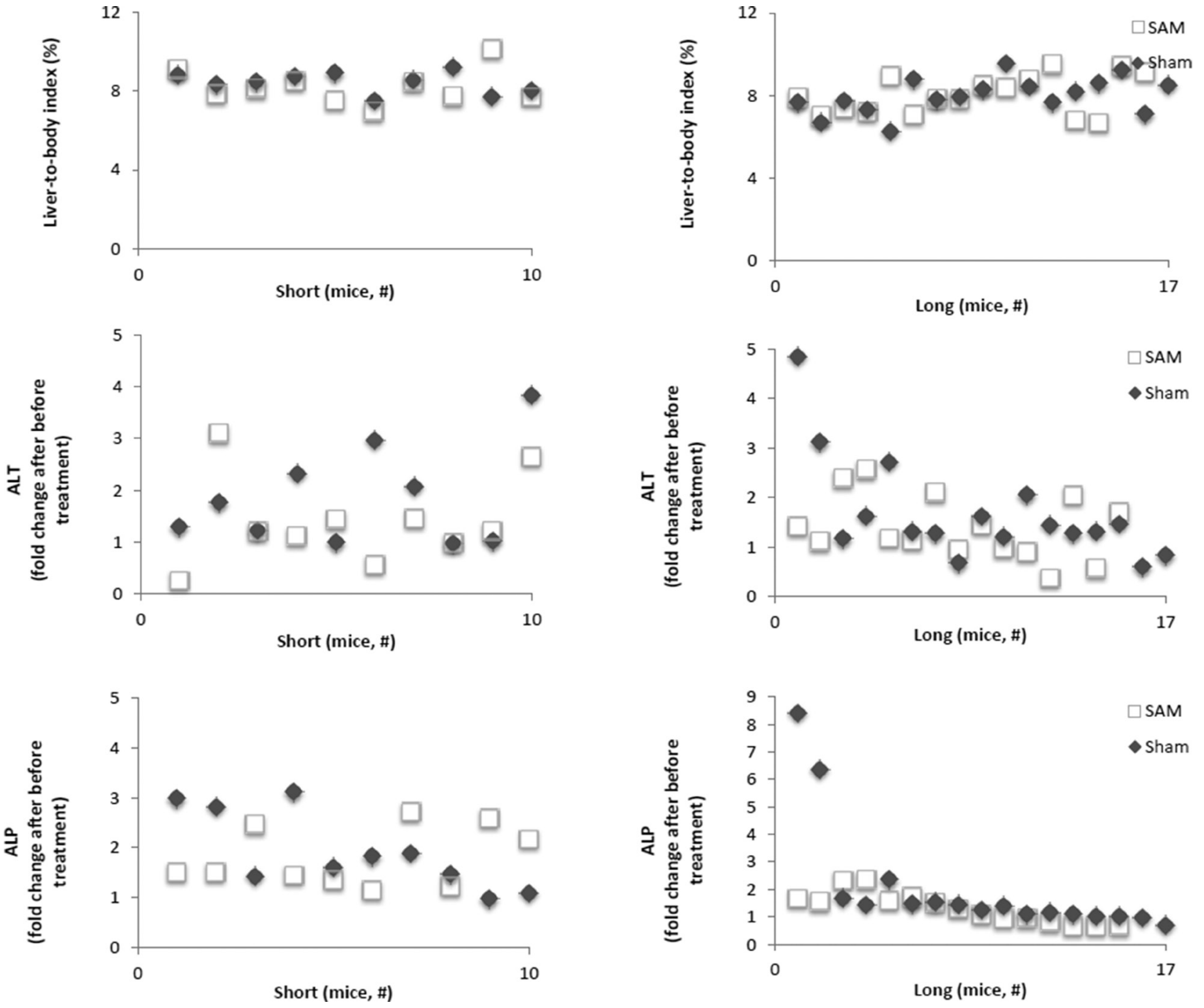

Supplementary Figure 4: Both the short-term (left panels) and the long-term (right panels) SAM treatments of Mdr2-KO mice did not change the liver-to body index or activities of liver enzymes in the serum. Upper panels – liver-to body index; middle panels – ALT activity; bottom panels – ALP activity. Ten males per experimental group.

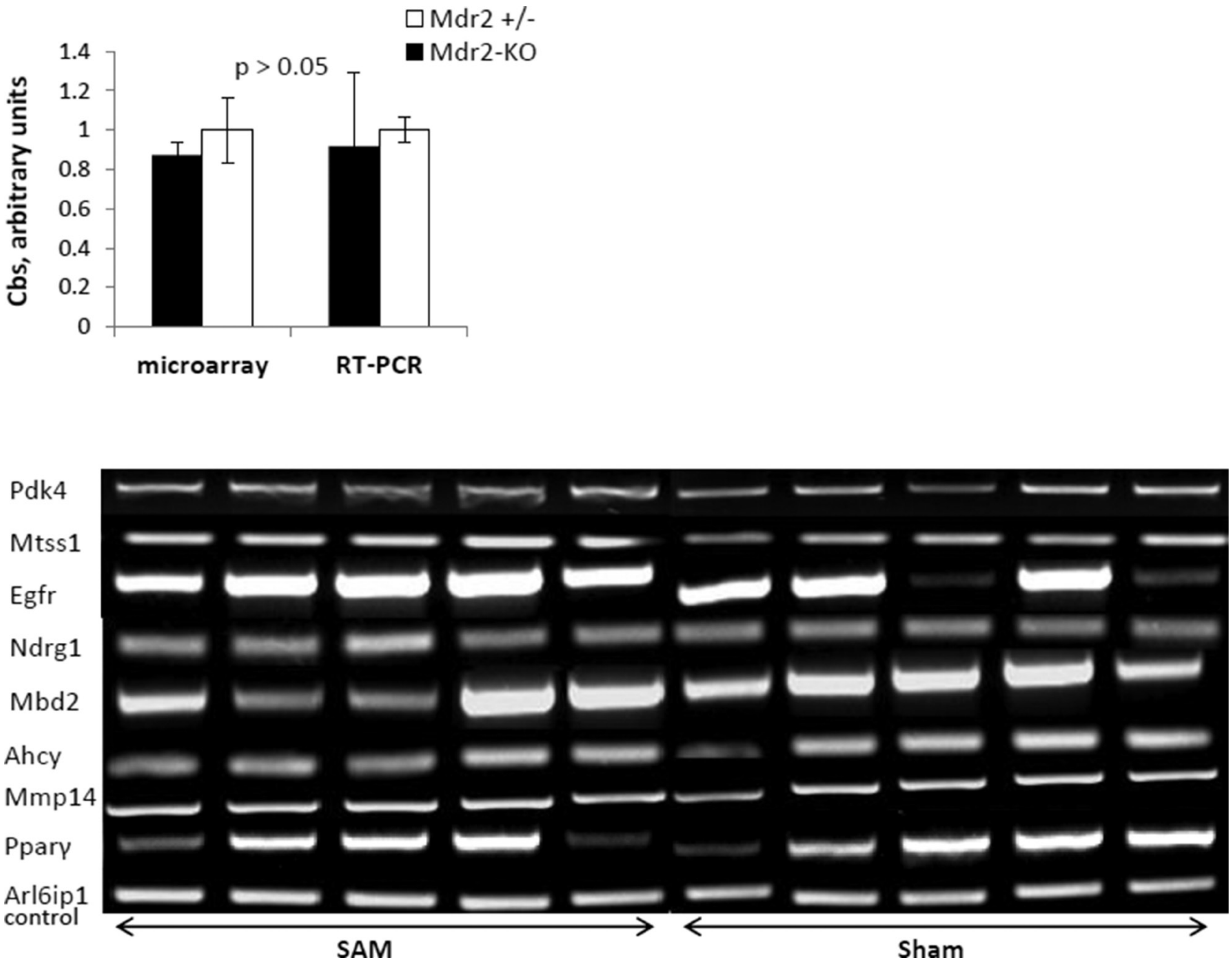

Supplementary Figure 5: Data on genes that did not change their expression level. Upper panel – quantitative RT-PCR demonstrated similar levels of the Cbs transcripts in the liver of Mdr2-KO and control Mdr2+/− mice. Bottom panel – semi-quantitative RT-PCR of genes that did not change significantly their expression following the short-term SAM treatment. Arl6ip1 – control “housekeeping” gene.

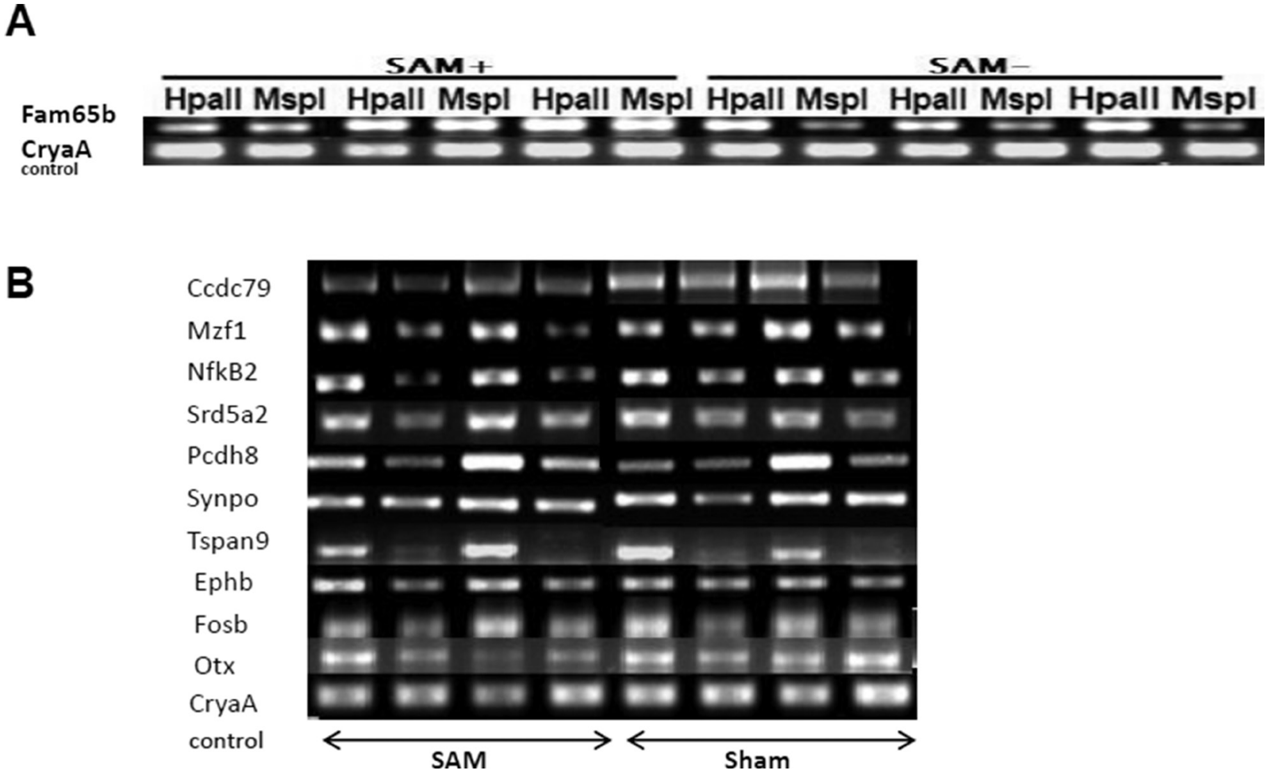

Supplementary Figure 6: Effect of the short-term SAM treatment on methylation of selected CpG islands that previously were shown to be hypermethylated in the liver of Mdr2-KO compared to control Mdr2+/− mice at the late precancerous stage (12-month-old). (**A**) SAM treatment restored the low methylation level of the CpG island in the Fam65b gene (quantitation – in the Figure 6B). (**B**) Genes that did not change methylation levels of their CpG islands following the short-term SAM treatment (shown in duplicates; however, three mice per experimental group were tested).

Supplementary Table 1: Gene expression in the liver of SAM-supplemented (SAM+) and sham-treated (SAM-) Mdr2-KO mice at the end of the short-term SAM supplementation, determined by the Nanostring assay (see Materials and Methods) [1]. See Supplementary_Table_1

Supplementary Table 2: Sequences of primers used for PCR. See Supplementary_Table_2

Supplementary Table 3: List of antibodies used in this study either for immunoblotting (IB) or for immunohistochemistry (IHC)

Antigene/antibody name	Manufacturer	Catalog number	Application	Dilution
βActin	Santa-Cruz, CA	Sc-1616	IB	1:200
p21/Cdkn1a	BD Bioscience	556430	IHC	1:1000
Anti-mouse	DAKO	K400411	IB, IHC	1:200
Anti-goat	DAKO	F025002	IB, IHC	1:200
Anti-Rabbit	DAKO	K401111	IB, IHC	1:200
γH2AX	BD Pharmingen	556431	IHC	1:1000
BrdU	DAKO	Mo744	IHC	1:50
F4-80	Serotec Raleigh, NC	MCA497	IHC	1:500
Mat1a	Santa-Cruz, CA	Sc-28029	IB, IHC	1:200
Phospho-SAPK/JNK	Cell Signaling technology	9251	IB	1:250
β-catenin	BD Biosciences	610153	IHC	1:150
Cxcl14	Abcam	ab137541	IB	1:500
Ki67	Thermo Scientific	RM-9106	IHC	1:500
