# Supplementary material for: Tumor-suppressive effect of S-adenosylmethionine supplementation in a murine model of inflammation-mediated hepatocarcinogenesis is dependent on treatment longevity
Source: Oncotarget. 2017 May 30;8(62):104772–84. doi: 10.18632/oncotarget.18300 (PMC5739599; doi:10.18632/oncotarget.18300)
Supplement: Supplementary file 2 [file oncotarget-08-104772-s002.docx]

**Supplementary Table 2.** Sequences of primers used for PCR. Primers for expression are from different exons; primers for MSRE include the CCGG restriction site. All primers are of mouse origin. All other primers, excluding those for Taqman and SYBR RT-PCR, were designed using the Primer3 software [1].

| Gene | Forward | Reverse | PCR method* | PCR assay |
| --- | --- | --- | --- | --- |
| Ahcy | TCGATGTGGAGATTGATGTG | AGTGAACCCCAACAGGGTAT | SQ | gene expression |
| Arl6ip1 | GTGTTCGCTCGTTGATAACCG | CCCATCGAAGGACTTTGTCAG | SQ, SYBR | gene expression |
| Arl6ip1 | MM01274631_m1 | | TQ | gene expression |
| Bzw2 | TGCAGAAGGTTCAGGAATACTGC | CGCTCAGAACGTCGGCTT | SYBR | gene expression |
| Cbs | CTCACATCCTGGAGATGGAC | GGTGTCTCTGAAAGCCAAGA | SQ | gene expression |
| Cebpa | CGCAAGAGCCGAGATAAAGC | CACGGCTCAGCTGTTCCA | SYBR | gene expression |
| Cdkn1b | CCAGACGTAAACAGCTCCGAAT | CATTCAATGGAGTCAGCGATATG | SYBR | gene expression |
| Crem | AGGCAAATGACCATGGAAAC | GTGTATTGCCCCGTGCTAGT | SYBR | gene expression |
| Cxcl14 | ACGGGTCCAAGTGTAAGTGT | GCAAAGTCCTCTGCTGAAGT | SQ | gene expression |
| Dgkz | CAGATCAGCCCCTGGCATGGAGAC | CCCACCCCGCAGCTGGGAAA | SQ | gene expression |
| Egfr | TCCTGGAGGGGGAACCAAGGGA | GGCCAATCCCAAGGGCCACCA | SQ | gene expression |
| Egfr | CCCATCCTCTGCAATATGGATAC | TGGATCACATTTGGGGCAA | SYBR | gene expression |
| Fam65b | GGGCCCAATGGCATCATCCG | AAGGCCCGGTAGACCTCCTC | SYBR | gene expression |
| Gadd45b | TGTACTTTGCTCTTGGGGATCTT | AGTCACCGCCTGCATCTTCT | SYBR | gene expression |
| Hprt | GTTAAGCAGTACAGCCCCAAA | AGGGCATATCCAACAACAAACTT | SQ, SYBR | gene expression |
| Hprt | MM00446968_m1 | | TQ | gene expression |
| Id3 | MM00492575_s1 | | TQ | gene expression |
| Igfals | GAGGTACTGACGCTCAATGACAA | GACAGTTGCCGGAGAGATTCA | SYBR | gene expression |
| Il6 | ACCACTCCCAACAGACCTGTCTATA | CAGAATTGCCATTGCACAACTCT | SYBR | gene expression |
| Il1r1 | MM00434237_m1 | | TQ | gene expression |
| Mbd2 | CCGACATCCTGTCCCGGGCT | GCTGCACTGCACCGGAAGGG | SQ | gene expression |
| Mmp14 | CACGCCACTGCGCTTCCGAG | GTTGGGCCCATAGGCGGGGT | SQ | gene expression |
| Mtss1 | TGGGTGCAGGCCCTTTCCCT | GGCTTTGCCCAGTCCTTCCAGC | SQ | gene expression |
| Ndrg1 | TGTCCCGAGAGCTACATGAC | CTCTTGCAGGAGACCAGTGA | SQ | gene expression |
| Pdk4 | GCCAGCCTAGGTGGGCGTCA | CCGTGGCCCTCATGGCATTCTTG | SQ | gene expression |
| Per3 | GTGCACTTTGTCGACCTGCTT | GCACATTCATACTGCGAGGCT | SYBR | gene expression |
| Pparg | ACCAAGTGACTCTGCTCAAGTAT | TGAAGGCTCATGTCTGTCTC | SQ | gene expression |
| Stmn1 | CGGACCGAGCAGGGCTTTCCTT | GCCATCTGCGCCTCCCGGTT | SQ | gene expression |
| Ccdc79 | CCCAGCCTCCGGGTCCACCG | CGGGAGGCGTGCATGGTGCC | SQ | MSRE |
| Ephb3 | GTGCCCCGAGAACCTGCGAC | GATGCCGGCAGGTCGTTCCC | SQ | MSRE |
| Fam65b | AGCGCTGGGGCCACGACTGT | ACCACCTCGGCACCCACGCA | SQ | MSRE |
| Fosb | GCTGCTCGCTGCCGCTGGTG | AGCAGCCGAGACGCACCCCC | SQ | MSRE |
| Mzf1 | TGGATGGCGCTGGACCAGGC | GGCCTTTCGTGTGCCGCGAC | SQ | MSRE |
| Nfkb2 | CGGGACACCGATGCTGGCGA | TCCCGCTAGCAGAGCACGCG | SQ | MSRE |
| Otx1 | GCGAGCGGACAGACACGGGC | CCGCCGAGCACGCCTGCAAC | SQ | MSRE |
| Pcdh8 | ACGTCTCGGATGCGCACGAT | GCAGGTGGACTACGAGCGCC | SQ | MSRE |
| Srd5a2 | AACCAGGCTATGCGTGCGGG | GGCGCTCCATAAAGGGGCCC | SQ | MSRE |
| Synpo | CTGCGCAGGTGGCAAGGGCGA | GGCTCCCGCTTGGGTCCCCTTCA | SQ | MSRE |
| Tspan9 | GGTGCCGTAGAAGGCGCCAG | GCTCAGAACCCGTGCCCGAC | SQ | MSRE |
| CryaA | CATTCAGCATCCTTGGTTCA | GCAGCAGGTCGTACTCAAAA | SQ, SYBR | MSRE, control gene |

SQ- semi-quantitative PCR,

SYBR – real-time PCR with SYBR mixture,

TQ - real-time PCR with Taqman reaction mixture.

1. Rozen, S. and H.J. Skaletsky, *Primer3 on the WWW for general users and for biologist programmers*, in *Bioinformatics Methods and Protocols: Methods in Molecular Biology*, S. Krawetz and S. Misener, Editors. 2000, Humana Press: Totowa, NJ. p. 365-386.
