# Supplementary material for: Tumor-suppressive effect of S-adenosylmethionine supplementation in a murine model of inflammation-mediated hepatocarcinogenesis is dependent on treatment longevity
Source: Oncotarget. 2017 May 30;8(62):104772–84. doi: 10.18632/oncotarget.18300 (PMC5739599; doi:10.18632/oncotarget.18300)
Supplement: Supplementary file 3 [file oncotarget-08-104772-s003.docx]

**Supplementary Table 1.** Gene expression in the liver of SAM-supplemented (SAM+) and sham-treated (SAM-) Mdr2-KO mice at the end of the short-term SAM supplementation, determined by the Nanostring assay (see Materials and Methods) [1]. Genes Arl6ip1, Polrmt, Ppia, and Rps20 were used as housekeeping genes. Other genes were selected from the literature for their involvement in the formation of a pro-tumorigenic microenvironment.

| Mouse ID 🡪 | D1922 | D1923 | D1935 | D1943 | D1921 | D1936 | D1951 | D1953 | Average | Average | Fold change | STDEV | STDEV |  |  |
| --- | --- | --- | --- | --- | --- | --- | --- | --- | --- | --- | --- | --- | --- | --- | --- |
| Gene symbol | SAM+ | SAM+ | SAM+ | SAM+ | SAM- | SAM- | SAM- | SAM- | SAM+ | SAM- | SAM+/ SAM- | SAM+ | SAM- | ttest | Gene symbol |
| Arg1 | 74546 | 90727 | 72149 | 100422 | 66443 | 58375 | 87570 | 76685 | **84461** | **72268** | **1.17** | 13465 | 12657 | 0.24 | Arg1 |
| Arl6ip1 | 9431 | 8315 | 8752 | 7765 | 9063 | 9303 | 8011 | 8784 | **8566** | **8790** | **0.97** | 704 | 561 | 0.64 | Arl6ip1 |
| Ccl2 | 467 | 477 | 606 | 689 | 761 | 2803 | 1154 | 420 | **560** | **1284** | **0.44** | 107 | 1056 | 0.22 | Ccl2 |
| Ccl3 | 41 | 46 | 44 | 45 | 40 | 81 | 49 | 33 | **44** | **51** | **0.88** | 2 | 21 | 0.58 | Ccl3 |
| Ccl5 | 505 | 256 | 181 | 348 | 291 | 201 | 192 | 184 | **322** | **217** | **1.49** | 140 | 50 | 0.21 | Ccl5 |
| Ccl17 | 61 | 41 | 54 | 53 | 64 | 89 | 132 | 45 | **52** | **82** | **0.63** | 8 | 37 | 0.16 | Ccl17 |
| Ccl21a | 499 | 142 | 84 | 242 | 169 | 152 | 379 | 160 | **241** | **215** | **1.12** | 183 | 110 | 0.81 | Ccl21a |
| Ccl22 | 50 | 34 | 23 | 23 | 26 | 39 | 53 | 23 | **32** | **35** | **0.92** | 13 | 13 | 0.76 | Ccl22 |
| Ccr1 | 76 | 61 | 74 | 126 | 82 | 91 | 99 | 38 | **84** | **78** | **1.08** | 29 | 28 | 0.75 | Ccr1 |
| Ccr6 | 32 | 38 | 21 | 29 | 30 | 34 | 26 | 24 | **30** | **29** | **1.05** | 7 | 4 | 0.74 | Ccr6 |
| Cd4 | 95 | 104 | 74 | 64 | 91 | 140 | 75 | 109 | **84** | **104** | **0.81** | 19 | 28 | 0.29 | Cd4 |
| Cd8a | 115 | 18 | 26 | 75 | 91 | 43 | 25 | 23 | **59** | **46** | **1.29** | 45 | 32 | 0.65 | Cd8a |
| Cd36 | 6193 | 4725 | 4327 | 5399 | 5248 | 4105 | 7232 | 5146 | **5161** | **5433** | **0.95** | 818 | 1306 | 0.74 | Cd36 |
| Cd44 | 884 | 684 | 673 | 711 | 832 | 1124 | 919 | 631 | **738** | **876** | **0.84** | 99 | 205 | 0.27 | Cd44 |
| Cd274 | 155 | 164 | 121 | 154 | 145 | 152 | 125 | 125 | **148** | **137** | **1.09** | 19 | 14 | 0.36 | Cd274 |
| Cxcl1 | 2561 | 1577 | 1344 | 17678 | 1416 | 5097 | 2361 | 1296 | **5790** | **2542** | **2.28** | 7943 | 1768 | 0.46 | Cxcl1 |
| Cxcl10 | 163 | 116 | 104 | 152 | 121 | 332 | 171 | 117 | **134** | **185** | **0.72** | 28 | 101 | 0.36 | Cxcl10 |
| Cxcl14 | 5609 | 4981 | 7186 | 4534 | 5679 | 6103 | 1906 | 1727 | **5578** | **3854** | **1.45** | 1160 | 2360 | 0.24 | Cxcl14 |
| Cxcl16 | 1326 | 1240 | 1218 | 1257 | 1208 | 1518 | 1285 | 1033 | **1260** | **1261** | **1.00** | 47 | 201 | 0.99 | Cxcl16 |
| Defb1 | 375 | 346 | 528 | 324 | 548 | 373 | 430 | 338 | **393** | **422** | **0.93** | 92 | 92 | 0.67 | Defb1 |
| Foxp3 | 14 | 14 | 11 | 12 | 7 | 13 | 17 | 13 | **13** | **12** | **1.01** | 2 | 4 | 0.93 | Foxp3 |
| Ifnb1 | 4 | 1 | 1 | 2 | 6 | 1 | 0 | 2 | **2** | **2** | **0.81** | 1 | 3 | 0.80 | Ifnb1 |
| Ifng | 30 | 26 | 21 | 28 | 30 | 36 | 28 | 23 | **26** | **29** | **0.91** | 4 | 5 | 0.46 | Ifng |
| Il1a | 448 | 323 | 335 | 292 | 393 | 504 | 293 | 338 | **350** | **382** | **0.92** | 68 | 91 | 0.59 | Il1a |
| Il1r1 | 2415 | 2195 | 2495 | 2386 | 1670 | 1701 | 1769 | 1988 | **2373** | **1782** | **1.33** | 127 | 143 | 0.001 | Il1r1 |
| Il2 | 9 | 5 | 2 | 7 | 12 | 2 | 6 | 4 | **6** | **6** | **0.95** | 3 | 5 | 0.91 | Il2 |
| Il2ra | 12 | 14 | 11 | 9 | 12 | 8 | 9 | 19 | **11** | **12** | **0.95** | 2 | 5 | 0.84 | Il2ra |
| Il4 | 4 | 6 | 1 | 5 | 2 | 0 | 1 | 2 | **4** | **1** | **3.14** | 2 | 1 | 0.06 | Il4 |
| Il6 | 19 | 14 | 27 | 14 | 12 | 11 | 10 | 11 | **19** | **11** | **1.68** | 6 | 1 | 0.06 | Il6 |
| Il10 | 5 | 2 | 4 | 4 | 4 | 7 | 10 | 0 | **4** | **5** | **0.78** | 1 | 4 | 0.63 | Il10 |
| Inhbe | 2627 | 2337 | 1739 | 2165 | 1994 | 2823 | 2824 | 3157 | **2217** | **2699** | **0.82** | 371 | 496 | 0.17 | Inhbe |
| Lep | 26 | 20 | 20 | 17 | 20 | 23 | 27 | 24 | **21** | **24** | **0.88** | 4 | 3 | 0.26 | Lep |
| Lgals1 | 5139 | 4168 | 5035 | 4596 | 5113 | 4905 | 5575 | 4595 | **4735** | **5047** | **0.94** | 445 | 412 | 0.34 | Lgals1 |
| Lta | 14 | 12 | 11 | 9 | 12 | 10 | 10 | 12 | **11** | **11** | **1.02** | 2 | 1 | 0.85 | Lta |
| Ly96 | 906 | 952 | 966 | 809 | 1067 | 971 | 893 | 765 | **908** | **924** | **0.98** | 71 | 127 | 0.84 | Ly96 |
| Ncr1 | 14 | 69 | 47 | 15 | 17 | 51 | 48 | 63 | **36** | **45** | **0.81** | 26 | 19 | 0.62 | Ncr1 |
| Nos2 | 9 | 8 | 8 | 8 | 9 | 5 | 14 | 8 | **8** | **9** | **0.94** | 1 | 4 | 0.77 | Nos2 |
| Pdcd1 | 5 | 4 | 8 | 8 | 4 | 13 | 4 | 5 | **6** | **6** | **1.00** | 2 | 5 | 1.00 | Pdcd1 |
| Polrmt | 183 | 185 | 191 | 251 | 214 | 222 | 226 | 205 | **202** | **217** | **0.93** | 32 | 9 | 0.43 | Polrmt |
| Ppia | 77671 | 79220 | 78010 | 79755 | 77603 | 77620 | 77875 | 76992 | **78664** | **77522** | **1.01** | 986 | 375 | 0.07 | Ppia |
| Prf1 | 59 | 56 | 50 | 40 | 59 | 40 | 52 | 54 | **51** | **51** | **1.00** | 8 | 8 | 0.98 | Prf1 |
| Prl | 6 | 2 | 10 | 10 | 6 | 5 | 4 | 5 | **7** | **5** | **1.38** | 4 | 1 | 0.36 | Prl |
| Rps20 | 28769 | 28333 | 29101 | 28283 | 29175 | 28908 | 29942 | 30073 | **28621** | **29525** | **0.97** | 387 | 571 | 0.04 | Rps20 |
| Slpi | 123 | 159 | 106 | 190 | 106 | 131 | 142 | 91 | **144** | **118** | **1.23** | 38 | 23 | 0.27 | Slpi |
| Socs1 | 69 | 58 | 37 | 69 | 42 | 55 | 48 | 44 | **58** | **47** | **1.23** | 15 | 5 | 0.22 | Socs1 |
| Socs2 | 1137 | 537 | 923 | 1379 | 2506 | 920 | 1283 | 892 | **994** | **1401** | **0.71** | 357 | 758 | 0.37 | Socs2 |
| Socs3 | 144 | 110 | 51 | 582 | 82 | 182 | 157 | 74 | **222** | **124** | **1.79** | 243 | 54 | 0.46 | Socs3 |
| Spp1 | 4788 | 2974 | 3980 | 2464 | 4471 | 4606 | 2971 | 2110 | **3552** | **3539** | **1.00** | 1037 | 1207 | 0.99 | Spp1 |
| Stat1 | 2902 | 2469 | 2510 | 2986 | 2295 | 2586 | 2370 | 2359 | **2717** | **2403** | **1.13** | 265 | 127 | 0.08 | Stat1 |
| Tnf | 25 | 15 | 23 | 14 | 17 | 60 | 18 | 14 | **19** | **27** | **0.71** | 6 | 22 | 0.51 | Tnf |

1. Geiss GK, Bumgarner RE, Birditt B, Dahl T, Dowidar N, Dunaway DL, Fell HP, Ferree S, George RD, Grogan T, James JJ, Maysuria M, Mitton JD, et al. Direct multiplexed measurement of gene expression with color-coded probe pairs. Nat Biotechnol. 2008; 26:317-325.
